# *In Vitro* Primer-Based RNA Elongation and Promoter Fine Mapping of the Respiratory Syncytial Virus

**DOI:** 10.1128/JVI.01897-20

**Published:** 2020-12-09

**Authors:** Dongdong Cao, Yunrong Gao, Claire Roesler, Samantha Rice, Paul D'Cunha, Lisa Zhuang, Julia Slack, Anna Antonova, Sarah Romanelli, Bo Liang

**Affiliations:** a Department of Biochemistry, Emory University School of Medicine, Atlanta, Georgia, USA; University of Kentucky College of Medicine

**Keywords:** *in vitro*, polymerases, primer-based RNA elongation, promoter mapping, respiratory syncytial virus

## Abstract

Respiratory syncytial virus (RSV) is a nonsegmented negative-sense (NNS) RNA virus and shares a similar RNA synthesis strategy with other members of NNS RNA viruses, such as measles, rabies virus, and Ebola virus. RSV RNA synthesis is catalyzed by a multifunctional RNA-dependent RNA polymerase (RdRP), which is composed of a large (L) protein that catalyzes three distinct enzymatic functions and an essential coenzyme phosphoprotein (P). Here, we successfully prepared highly pure, full-length, wild-type and mutant RSV polymerase (L-P) complexes. We demonstrated that the RSV polymerase could carry out both *de novo* and primer-based RNA synthesis. We defined the minimal length of the RNA template for *in vitro de novo* RNA synthesis using the purified RSV polymerase as 8 nucleotides (nt), shorter than previously reported. We showed that the RSV polymerase catalyzed primer-dependent RNA elongation with different lengths of primers on both short (10-nt) and long (25-nt) RNA templates. We compared the sequence specificity of different viral promoters and identified positions 3, 5, and 8 of the promoter sequence as essential to the *in vitro* RSV polymerase activity, consistent with the results previously mapped with the *in vivo* minigenome assay. Overall, these findings agree well with those of previous biochemical studies and extend our understanding of the promoter sequence and the mechanism of RSV RNA synthesis.

**IMPORTANCE** As a major human pathogen, RSV affects 3.4 million children worldwide annually. However, no effective antivirals or vaccines are available. An in-depth mechanistic understanding of the RSV RNA synthesis machinery remains a high priority among the NNS RNA viruses. There is a strong public health need for research on this virus, due to major fundamental gaps in our understanding of NNS RNA virus replication. As the key enzyme executing transcription and replication of the virus, the RSV RdRP is a logical target for novel antiviral drugs. Therefore, exploring the primer-dependent RNA elongation extends our mechanistic understanding of the RSV RNA synthesis. Further fine mapping of the promoter sequence paves the way to better understand the function and structure of the RSV polymerase.

## Abstract (precis)

As a major human pathogen, RSV affects 3.4 million children worldwide annually. However, no effective antivirals or vaccines are available. An in-depth mechanistic understanding of the RSV RNA synthesis machinery remains a high priority among the NNS RNA viruses. There is a strong public health need for research on this virus, due to major fundamental gaps in our understanding of NNS RNA virus replication. As the key enzyme executing transcription and replication of the virus, the RSV RdRP is a logical target for novel antiviral drugs. Therefore, exploring the primer-dependent RNA elongation extends our mechanistic understanding of the RSV RNA synthesis. Further fine mapping of the promoter sequence paves the way to better understand the function and structure of the RSV polymerase.

## INTRODUCTION

Respiratory syncytial virus (RSV) infection is a leading cause of severe lower track respiratory diseases in young children, older adults, and immunocompromised individuals in the United States and worldwide (1, 2). Currently, there are no effective vaccines or antivirals available to prevent or to treat RSV infection (3–6). As a significant human pathogen of the nonsegmented negative-sense (NNS) RNA viruses, RSV shares a common strategy for genome replication and gene expression with other NNS RNA viruses, such as measles, rabies virus (RABV), and Ebola virus (EBOV) (7–11). RSV RNA synthesis is catalyzed by a multifunctional RNA-dependent RNA polymerase (RdRP), which is composed of a large protein (L) that catalyzes three distinct enzymatic functions and an essential coenzyme phosphoprotein (P) (12–17). The polymerases catalyze both replication of viral genomes and transcription of viral genes, and RNA synthesis is central to NNS RNA viral life cycles.

The RSV genome contains 10 genes that encode 11 proteins (1, 2). The RSV negative-sense genome acts as the template for replication to produce positive-sense antigenomic RNA, as well as for transcription to synthesize 10 viral mRNAs. The antigenome, in turn, becomes the template to generate genomic RNA (1, 17, 18). Both the genomic and antigenomic RNAs are encapsidated directly by the nucleoprotein (N) during their synthesis, and each N protein binds 7 nucleotides (nt) (19). Those encapsidated RNAs remain coated during RNA synthesis (1, 2). NNS RNA synthesis is thought to follow the “start-stop model” of sequential and polar transcription (20, 21). Recent studies also suggested alternative gene expression strategies using nongradient and genotype-dependent transcription in RSV and EBOV (22, 23). During transcription, using a single promoter in the 3′ leader (Le) region of the genome, the polymerase initiates and terminates mRNA transcription in response to the gene start (GS) and gene end (GE) signals. During replication, with the promoters at the 3′ ends of the genomic or antigenomic RNAs, the polymerase ignores all GS and GE signals to synthesize full-length cRNAs (24, 25). The 44-nt Le sequence at the 3′ end of the genome and the 155-nt trailer complementary (TrC) sequence at the 3′ end of the antigenome serve as the promoters for RNA synthesis (26, 27) (see Fig. 2A).

Previous studies by others of the NNS viral polymerase activities and the genomic and antigenomic promoters using cell-based minigenome assays and *in vitro* assays with recombinant polymerase have provided insights regarding NNS RNA synthesis (25–42). For example, Collins and colleagues pioneered RNA element mapping using *in vivo* cell-based minigenome assays (25, 28). Fearns and colleagues demonstrated the ability of polymerase to initiate *de novo* RNA synthesis at positions 1 and 3 (34, 35) and revealed that the template RNA was able to fold into a secondary structure to extend at the 3′ end using a back-priming mechanism (34). Whelan and colleagues pioneered the *in vitro* characterization of RNA synthesis of vesicular stomatitis virus (VSV) and RABV polymerases (36, 37). Ogino and colleagues established the unconventional mRNA capping assay for *Rhabdoviruses* (41, 42). Recently, Gotte and Deval and colleagues showed primer-dependent elongation using an *in vitro* assay for EBOV and Nipah virus (NiV) polymerases (38, 39). Deval et al. also showed primer-dependent elongation for RSV using a short (11-nt) mutant RNA template (40). Together, those findings provided valuable platforms and protocols for in-depth mechanistic analyses of RSV RNA synthesis.

Here, we successfully prepared the high-quality RSV polymerase (L-P) complex and its catalytic mutant, which allowed us to perform in-depth structural and biochemical analyses. We integrated and adapted the RNA polymerization assays from protocols developed for RSV and other NNS RNA viruses (34–40). We used naked RNAs as the templates to study *in vitro* RNA synthesis. We demonstrated that the RSV polymerase could carry out both *de novo* and primer-dependent RNA synthesis. We defined the minimal length of the RNA template for *de novo* RNA synthesis of the RSV polymerase as 8 nt, shorter than previously reported (33, 34, 38). We showed that the RSV polymerase catalyzes primer-dependent RNA elongation with different lengths of primers on both short (10-nt) and long (25-nt) RNA templates. We compared the sequence specificity of different viral promoters and identified positions 3, 5, and 8 of the promoter sequence as being essential for *in vitro* RSV polymerase activity, consistent with the results previously mapped by the *in vivo* minigenome assay (27). This work agreed with previous biochemical studies and provided new mechanistic insights into the initiation and elongation of RSV RNA synthesis (25–42).

## RESULTS

### Preparation and molecular architecture of the RSV polymerase.

**FIG 1:**
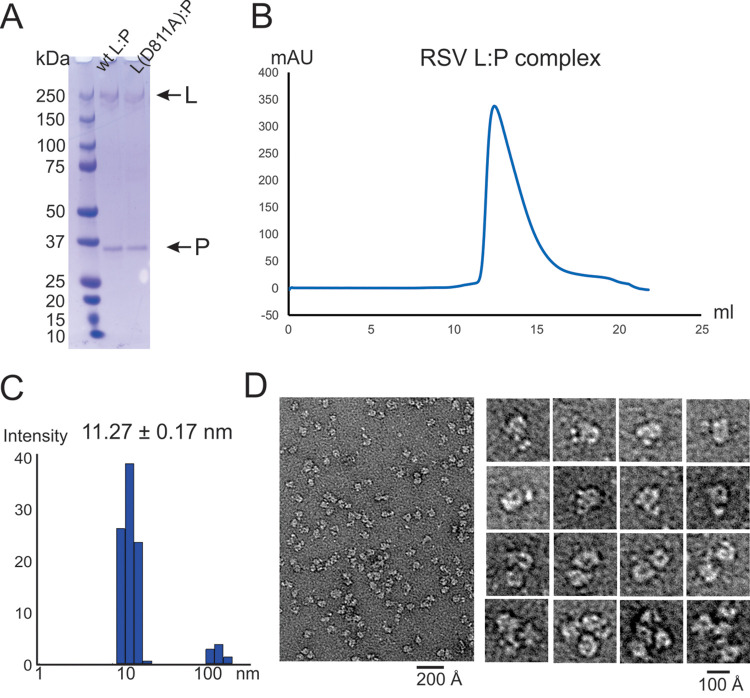
Preparation and molecular architecture of RSV polymerase. (A) SDS-PAGE analysis of the purified recombinant wt and catalytic mutant (D811A) RSV polymerase (L-P complex). (B) Elution profile of representative size exclusion chromatography for the RSV L-P complex. (C) DLS data for the RSV polymerase (Rh = 17.8 ± 0.5 nm). The DLS experiments were performed in triplicate. (D) A representative negative-stain EM image of the RSV polymerase (L-P complex) is shown on the left (scale bar, 200 Å). The insets on the right are magnified representative views of the raw image. The majority population of the particles is shown in the upper insets as monomeric species of the polymerase, and the minor population of the particles is shown in the lower insets as higher oligomeric species of the polymerase.

To enable in-depth mechanistic analyses of RSV RNA synthesis, preparation of high-quality RSV polymerase was the critical first step. The P protein plays a critical role in stabilizing the RSV L protein, and it is necessary to coexpress L and P proteins together (43). To obtain the RSV polymerase (L-P complex), we optimized and revised the protocol initially established by Fearns and colleagues (34). Briefly, we subcloned the codon-optimized RSV L and P in a pFastBac Dual vector and coexpressed recombinant L-P complex in Sf21 insect cells using baculoviruses. For purification purposes, we engineered an N-terminal 6×His tag and tobacco etch virus (TEV) protease cleavage sequence on the RSV L protein, with the RSV P protein being tag free. We isolated the RSV L-P complex using Co^2+^-nitrilotriacetic acid (NTA), removed the 6×His tag through TEV protease cleavage, and then ran the sample through a heparin column, followed by size exclusion chromatography. We used this procedure to prepare mostly pure wild-type (wt) L-P complex in good quantity. Previous studies reported that HSP70 or HSC70 usually comigrates with L-P complex (34, 44). In this study, we eliminated these contaminants using the revised protocol for preparation (Fig. 1A). We also purified the catalytically inactive mutant L(D811A) with a similar strategy. The size exclusion chromatography profile shows a monodisperse peak of the RSV L-P complex (Fig. 1B). The purified RSV polymerase (L-P) has also been successfully used for high-resolution cryo-electron microscopy (EM) analysis (45).

Next, we used dynamic light scattering (DLS) to determine the size distribution of the L-P complex. Histogram analysis revealed that the purified L-P complex had a relatively narrow peak with a measured hydrodynamic radius (Rh) of 11.27 ± 0.17 nm (98.5%), suggesting a homogeneous conformational distribution of L-P (Fig. 1C). The RSV P contains an oligomeric domain, and P is a tetrameric protein in solution (46). We speculated that the L-P complex forms higher oligomeric states, such as a dimer (similar to the VSV L-P) or tetramer, due to the oligomerization domain of P (47, 48). We used negative-stain EM to image the RSV L-P complex. The images showed individual particles of the RSV polymerase on the grid. Interestingly, the majority of L-P complex particles from the peak were a single species rather than higher oligomeric particles. Those single-species particles agree well with the recent high-resolution cryo-EM structures of the RSV polymerase (45, 49). The magnified images of monomeric L-P show a ring-like core domain plus a few appendages, with views similar to that of VSV L-P (48, 50) (Fig. 1D, upper insets). As expected, we also observed a small fraction of samples presumably forming higher oligomers, such as dimers, trimers, and tetramers (Fig. 1D, lower insets). Consistent with the gel filtration and DLS results, negative-stain EM images showed a largely pure and monomeric population of polymerases.

### *In vitro* reconstitution of *de novo* RSV RNA synthesis.

**FIG 2:**
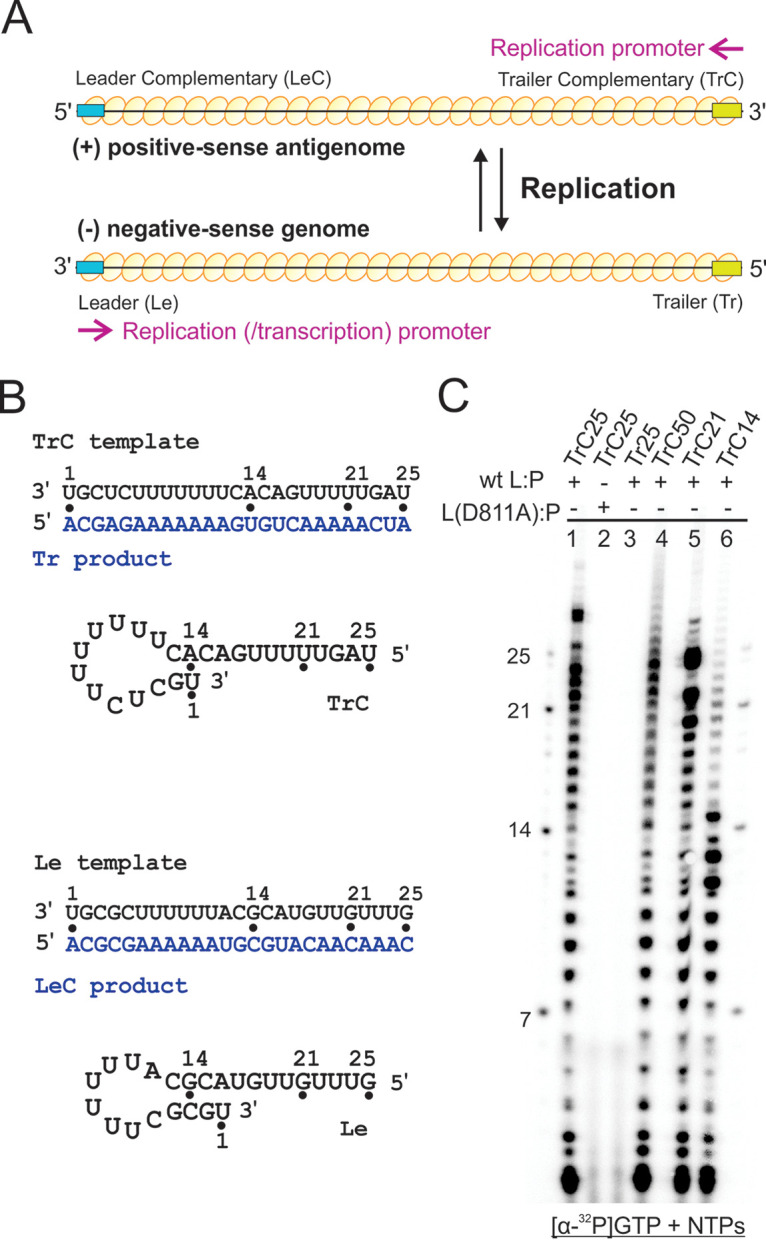
*In vitro* reconstitution of RSV RNA synthesis. (A) Illustrations of RNA synthesis by the RSV polymerase. Le or LeC and Tr or TrC are indicated using cyan and gold boxes, respectively. The locations of the promoter RNA with respect to the N protein (yellow oval)-encapsidated genome and antigenome are indicated. (B) Sequences and secondary structures of the RSV Le and TrC RNA templates (black text) and the Tr and LeC products (blue text). Positions 7, 14, 21, and 25 are highlighted using black dots. The secondary structures are predicted using mFold. Note the base-pairing differences between the TrC and Le templates. (C) *In vitro* RNA synthesis assays of various lengths and sequences of RNA templates by the RSV polymerase (wt and mutant). The reaction mixtures were incubated with NTPs (ATP, CTP, and UTP each at 1.25 mM and GTP at 50 μM with 5 μCi of [α-^32^P]GTP). The wt RSV polymerase is shown in lanes 1 and 3 to 6, and the mutant RSV polymerase is shown in lane 2. The products were analyzed using a 20% polyacrylamide gel containing 7 M urea, followed by phosphorimaging. The left and right lanes show the molecular weight ladder. The sequences of the RNA oligonucleotides are listed in Table 1.

To illustrate the locations of the promoter RNAs with respect to the N protein (Fig. 2A, yellow oval)-encapsidated genome and antigenome, we highlighted the Le or Le complementary (LeC) and trailer (Tr) or TrC sequences (Fig. 2A, cyan and gold boxes, respectively). To reconstitute *in vitro* RSV RNA synthesis, we adapted the protocols developed for RSV and other NNS RNA viruses (34–39). Briefly, the RSV polymerase (L-P) was incubated with the RNA oligonucleotides in the presence of one or four nucleoside triphosphates (NTPs) and α-^32^P-labeled GTP, ATP, or UTP. The lengths of the RNA ladder were 7 nt, 14 nt, 21 nt, and 25 nt. The sequences of the RNA oligonucleotides are listed in Table 1. Because the RNA oligonucleotides we used in our assays were naked RNAs (not encapsidated by the N proteins), we also calculated and plotted the lowest energy of potential secondary structures using mFold (51) (Fig. 2B).

**TABLE 1:** RNA oligonucleotides used in this study

Name	RNA oligonucleotide sequence
RSV-Le19	3′-UGCGCUUUUUUACGCAUGU
RABV-Le19	3′-UGCGAAUUGUUGGUCUAGU
VSV-Le19	3′-UGCUUCUGUUUGUUUGGUA
NiV-Le19	3′-UGGUUUGUUCCCUCUUAUA
EBOV-Le19	3′-GCCUGUGUGUUUUUCUUUC
RSV-TrC19	3′-UGCUCUUUUUUUCACAGUU
Le14	3′-UGCGCUUUUUUACG
LeC14	3′-CGUAAAAAAGCGCA
TrC7	3′-UGCUCUU
TrC8	3′-UGCUCUUU
TrC9	3′-UGCUCUUUU
TrC10	3′-UGCUCUUUUU
TrC11	3′-UGCUCUUUUUU
TrC12	3′-UGCUCUUUUUUU
TrC13	3′-UGCUCUUUUUUUC
TrC14	3′-UGCUCUUUUUUUCA
TrC21	3′-UGCUCUUUUUUUCACAGUUUU
TrC25	3′-UGCUCUUUUUUUCACAGUUUUUGAU
TrC25−3	3′-UCUUUUUUUCACAGUUUUUGAU
TrC25−2	3′-CUCUUUUUUUCACAGUUUUUGAU
TrC25−1	3′-GCUCUUUUUUUCACAGUUUUUGAU
TrC25+1	3′-GUGCUCUUUUUUUCACAGUUUUUGAU
TrC25+2	3′-UGUGCUCUUUUUUUCACAGUUUUUGAU
TrC50	3′-UGCUCUUUUUUUCACAGUUUUUGAUUAUAGAGCAUUAAAUCAAUUAUGUG
Tr7	5′-ACGAGAA
Tr14	5′-ACGAGAAAAAAAGU
Tr21	5′-ACGAGAAAAAAAGUGUCAAAA
Tr25	3′-AUCAAAAACUGUGAAAAAAAGAGCA
p2	5′-AC
p3	5′-ACG
p4	5′-ACGA
p5	5′-ACGAG
p7	5′-ACGACAA
p5*	5′-UAGUU
p5̂	5′-ACGCG
p5#	5′-GCAUU
TrC10(wt)	3′-UGCUCUUUUU
TrC10(U1G)	3′-GGCUCUUUUU
TrC10(U1C)	3′-CGCUCUUUUU
TrC10(U1A)	3′-AGCUCUUUUU
TrC10(G2C)	3′-UCCUCUUUUU
TrC10(G2A)	3′-UACUCUUUUU
TrC10(G2U)	3′-UUCUCUUUUU
TrC10(C3G)	3′-UGGUCUUUUU
TrC10(C3A)	3′-UGAUCUUUUU
TrC10(C3U)	3′-UGUUCUUUUU
TrC10(U4G)	3′-UGCGCUUUUU
TrC10(U4C)	3′-UGCCCUUUUU
TrC10(U4A)	3′-UGCACUUUUU
TrC10(C5G)	3′-UGCUGUUUUU
TrC10(C5A)	3′-UGCUAUUUUU
TrC10(C5U)	3′-UGCUUUUUUU
TrC10(U6G)	3′-UGCUCGUUUU
TrC10(U6C)	3′-UGCUCCUUUU
TrC10(U6A)	3′-UGCUCAUUUU
TrC10(U7G)	3′-UGCUCUGUUU
TrC10(U7C)	3′-UGCUCUCUUU
TrC10(U7A)	3′-UGCUCUAUUU
TrC10(U8G)	3′-UGCUCUUGUU
TrC10(U8C)	3′-UGCUCUUCUU
TrC10(U8A)	3′-UGCUCUUAUU
TrC12(12C)	3′-UGCUCUUUUUUC
TrC12(10–12C)	3′-UGCUCUUUUCCC
TrC12(8–12C)	3′-UGCUCUUCCCCC
TrC12(6–12C)	3′-UGCUCCCCCCCC
TrC12(6–11C)	3′-UGCUCCCCCCCU

We showed that the RSV polymerase has polymerase activity on a nonencapsidated RNA corresponding to the first 25 nt of the 3′ end of the TrC sequence (TrC25) (Fig. 2C, lane 1). Radioactively labeled products ranging from 2 to 25 nt in length were detected, with an additional band at 27/28 nt. This result demonstrates that the RSV polymerase synthesizes RNA *in vitro*, although the polymerase is poorly processive. A previous study by Fearns and colleagues suggested that the other larger bands were due to self-priming of the TrC25 template (34). As expected, no products were detected in reaction mixtures containing the catalytically inactive L(D811A) mutant (Fig. 2C, lane 2). This finding confirms that the RNA synthesis activity was abolished from the RSV L(D811A) mutant, similar to the previously reported RSV L(N812A) mutant (34). We investigated the sequence properties of the template in the RNA synthesis activities of L. Reaction mixtures containing the RNA template of the complement of the promoter sequence (Tr25) did not generate RNA products, suggesting promoter specificity of the RSV polymerase (Fig. 2C, lane 3).

Further, we checked several RNA templates with varied lengths and sequences. The RSV polymerase also synthesized RNA for longer (TrC50) and shorter (TrC21 and TrC14) RNA templates, as shown in abortive products (Fig. 2C, lanes 4 to 6). A ladder of abortive products was generated also for TrC50, gradually diminishing after 25 nt. The longest product was weak and less than 30 nt, far from the length of the template (50 nt). For TrC21, the products ranged from 2 to 21 nt, with additional bands of 23 nt or more. Interestingly, products of TrC14 were generated up to 25 nt, about 10 nt longer than the template. It is possible that the polymerase adds extra As by stuttering on the sequence of U6–13. In summary, the prepared RSV polymerase was active and could work on templates of different lengths, similar to previously reported studies (32, 35, 52, 53). For simplicity, we used TrC RNA templates with lengths of ≤25 nt for most of the experiments in this study.

### Template length requirements for *de novo* RNA synthesis.

**FIG 3:**
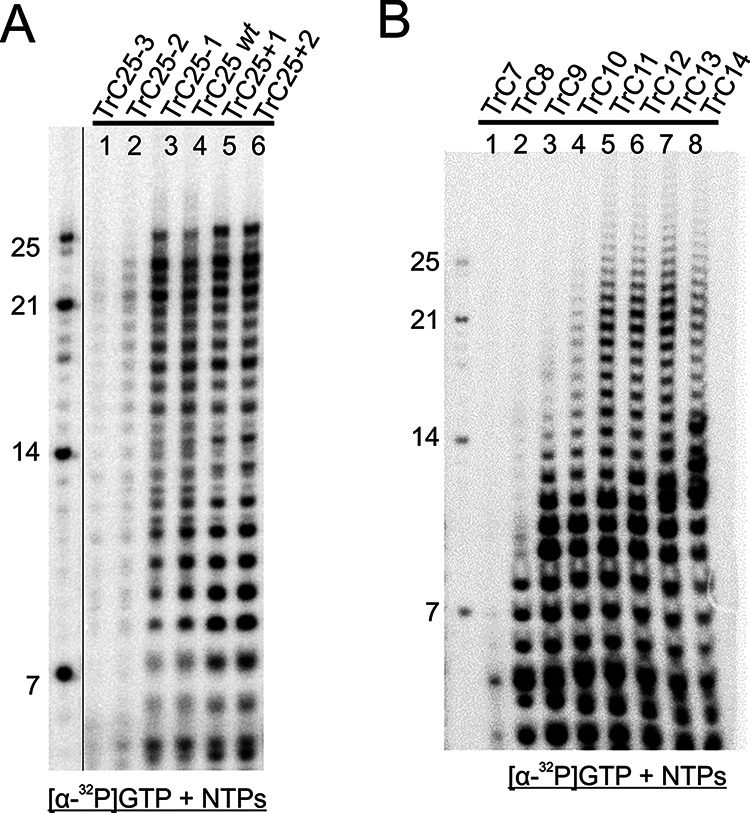
Template length requirements for RSV RNA synthesis. (A) TrC25 and variants were examined in the presence of NTPs (ATP, CTP, and UTP each at 1.25 mM and GTP at 50 μM with 5 μCi of [α-^32^P]GTP) (lanes 1 to 6). The deletion and addition of the 3′ end of the RNA template based on wt TrC25, namely, TrC25−3, TrC25−2, TrC25−1, TrC25+1, and TrC25+2, were used. The left lane shows the molecular weight ladder. The sequences of the RNA oligonucleotides are listed in Table 1. (B) TrC7, TrC8, TrC9, TrC10, TrC11, TrC12, TrC13, and TrC14 were used to define the minimal length requirements for the RSV polymerase activities. The reaction mixtures were incubated in the presence of NTPs (ATP, CTP, and UTP each at 1.25 mM and GTP at 50 μM with 5 μCi of [α-^32^P]GTP). The left lane shows the molecular weight ladder. The sequences of the RNA oligonucleotides are listed in Table 1.

Having shown that the RSV polymerase was active, we characterized the template length requirements for RSV RNA synthesis. First, to further investigate the specificity of the promoter sequence, we examined the polymerase activities with the addition or deletion of the first nucleotide(s) at the 3′ terminus of the promoter sequence. Previous studies indicated that the first nucleotides of the promoter sequence are critical for the polymerase to initiate RNA synthesis (27, 31). We prepared variations (−3, −2, −1, +1, and +2) at the 3′ end of TrC25(wt) (Table 1). We used both [α-^32^P]GTP and [α-^32^P]ATP to compare the polymerase activity (Fig. 3; also see Fig. S1 in the supplemental material). Interestingly, the results of both isotope labeling showed that, for deletions, −1 did not lose most activity (Fig. 3A, lane 3) and −2 and −3 lost at least one-half of the polymerase activity, compared to TrC25(wt) (Fig. 3A, lanes 1 and 2). The addition of nucleotides, +1 and +2, did not reduce the polymerase activity but rather enhanced the polymerase activity in the case of [α-^32^P]GTP (Fig. 3A, lanes 5 and 6) and maintained similar levels in the case of [α-^32^P]ATP (Fig. S1A).

Next, we examined the minimum length requirement of the RNA templates for RNA synthesis by the RSV polymerase. Based on our results, we knew that TrC14 could serve as the template. We further trimmed the size of the RNA and tested whether TrC13, TrC12, or shorter templates represented the minimal lengths. The results showed that TrC7 could not generate RNA products (Fig. 3B, lane 1) but TrC8 and TrC9 could make partial products with reduced polymerase activity (Fig. 3B, lanes 2 and 3). TrC10 and larger (TrC11, TrC12, and TrC13) could make regular RNA products (Fig. 3B, lanes 4 to 8). Therefore, we concluded that the minimal length of the template for RSV RNA synthesis is 8 nt. We compared the polymerase activity with the same set of templates using [α-^32^P]ATP (Fig. S1B), and it seemed that [α-^32^P]GTP gave stronger signals. This may be due to the specific sequences to be added to the nascent RNA products. For simplicity, we focused on TrC10 as the minimum template for effective *de novo* initiation and used [α-^32^P]GTP as the labeling nucleotide for further testing.

### RNA template specificity for *de novo* RNA synthesis by the RSV polymerase.

**FIG 4:**
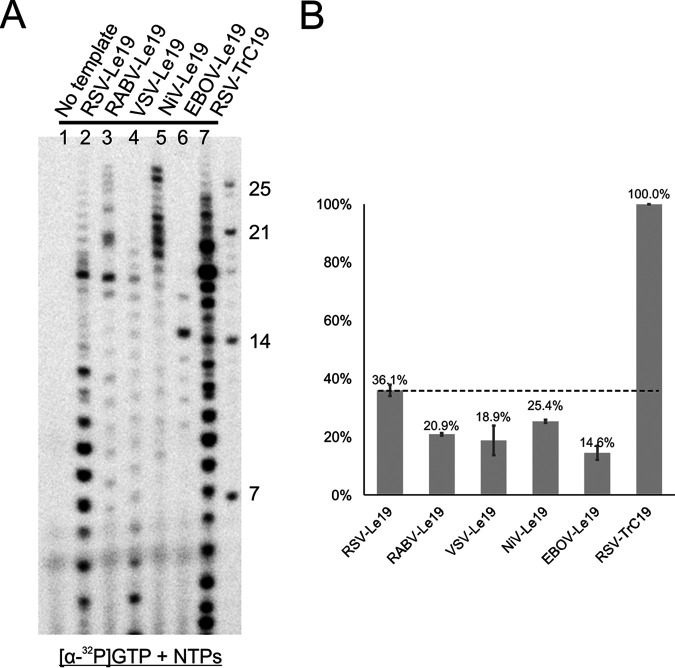
RNA template specificity for *de novo* RNA synthesis by the RSV polymerase. (A) Le sequences (19 nt) from RSV, RABV, VSV, NiV, and EBOV and TrC19 from RSV were used in *de novo* RSV polymerase assays. The reaction mixtures were incubated in the presence of NTPs (ATP, CTP, and UTP each at 1.25 mM and GTP at 50 μM with 5 μCi of [α-^32^P]GTP). The products of the reaction mixtures were shown for control (no template), RSV-Le19, RABV-Le19, VSV-Le19, NiV-Le19, EBOV-Le19, and RSV-TrC19. The right lane shows the molecular weight ladder. The sequences of the RNA oligonucleotides are listed in Table 1. (B) Total polymerase activities from panel A were quantified and plotted. The sample size was *n* = 2, and the error bars represent the standard deviation. The dashed line indicates the polymerase activities of RSV-Le19.

The RNA synthesis products of each template revealed a specific pattern, showing an accumulation of products predominantly around positions 8, 9, and 10 when the templates were copied. To further characterize the RNA template specificity, we compared the activities of the RSV polymerase using Le19 from RSV, RABV, VSV, NiV, and EBOV, as well as TrC19 from RSV (Table 1). As expected, the RSV-TrC19 template showed the highest polymerase activity (Fig. 4A, lane 7), while RSV-Le19 showed the second-highest polymerase activity (Fig. 4A, lane 2), compared to Le19 from other viruses (Fig. 4A, lanes 3 to 6). When quantified and normalized RNA synthesis activities were compared, assuming RSV-TrC19 as 100%, RSV-Le19 activity (36.1%) was the second highest (Fig. 4B, dashed line) and was much higher than that of RABV-Le19 (20.9%), VSV-Le19 (18.9%), NiV-Le19 (25.4%), and EBOV-Le19 (14.6%) (Fig. 4B).

It is interesting to note that several longer RNA oligonucleotides but not shorter RNA oligonucleotides were generated using NiV-Le19 and RABV-Le19. When we checked the potential self-dimer based on the sequence secondary structure prediction, both RNA oligonucleotides formed a partially overlapped duplex. It is possible that the longer RNA bands generated represented extension of the RNA templates using a back-priming mechanism. Together, although a uridine-rich sequence is commonly found in the 3′ end of those NNS RNA viruses, our results suggest that the specificity of the RNA template resides in the specific promoter sequence.

### RNA elongation using a primed RNA template (primer-dependent elongation).

**FIG 5:**
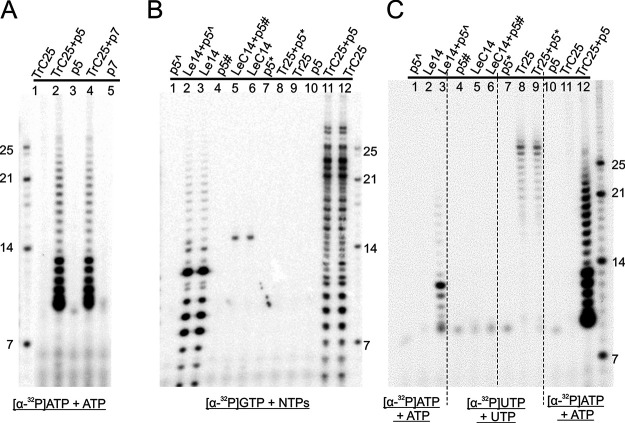
RNA elongation using a primed RNA template. (A) A 25-nt template (TrC25) with 5 or 7 bases complementary to a short 5-nt/7-nt primer (p5/p7) was used to analyze primer elongation activity of the RSV polymerase. The reaction mixtures were incubated in the presence of ATP only (ATP at 50 μM with 5 μCi of [α-^32^P]ATP). The left lane shows the molecular weight ladder. The sequences of the RNA oligonucleotides are listed in Table 1. TrC25 with both p5 and p7 primers but not TrC25 without primers could be elongated by the RSV polymerase. (B) The RNA templates were incubated with their 5-nt primers in the presence of NTPs (ATP, CTP, and UTP each at 1.25 mM and GTP at 50 μM with 5 μCi of [α-^32^P]GTP). The right lane shows the molecular weight ladder. The sequences of the RNA oligonucleotides are listed in Table 1. The products are shown using denaturing RNA gel. As expected, there was no significant difference between the templates with and without primers. (C) The same RNA templates were incubated with their respective 5-nt primers in the presence of ATP (ATP at 50 μM with 5 μCi of [α-^32^P]ATP) or UTP (UTP at 50 μM with 5 μCi of [α-^32^P]UTP). The right lane shows the molecular weight ladder. The sequences of the RNA oligonucleotides are listed in Table 1. The Le14+p5̂ worked as expected but not Le14 or p5̂ alone.

Previous studies reported primer-dependent elongation on a short (11-nt) mutant RNA template (33, 54, 55). We further tested whether the RSV polymerase could carry out RNA synthesis on longer (25-nt and 14-nt) primed templates. We examined all terminal sequences for RSV using a complementary 5-nt or 7-nt primer (sequences in Table 1). Because of the RNA template and primer sequences, we used [α-^32^P]ATP and ATP only for TrC25. We preannealed the primer with TrC25 and then incubated it with the RSV polymerase. As expected, TrC25 itself did not generate any product (Fig. 5A, lane 1), and neither did the p5/p7 primers themselves (Fig. 5A, lanes 3 and 5). Interestingly, when we incubated both TrC25 and p5/p7, we could readily detect products ranging from 9 to 13 nt, as well as weaker bands longer than the template (Fig. 5A, lanes 2 and 4). These results revealed that (i) the RSV polymerase is capable of extending the RNA transcript when a primer is provided and (ii) the first abortive product is 9 nt long, rather than 6 nt in the case of p5 or 8 nt in the case of p7. It is possible that the initial elongation to 9 nt is highly processive but, after reaching 9 nt, the products become less processive and generate abortive products at every position (Fig. 5A). It is also possible that the 5′ end of the primers contains an OH group and the migration pattern was due to the chemical nature of the primer, similar to findings described previously (55). Interestingly, these results seemed different from others obtained using primers with a short (11-nt) template, for which the polymerase elongated RNA continuously after the primers (33, 38). One possible explanation could be that the lengths of RNA template (25 nt or 14 nt) used in this study are longer than the 11 nt used previously (33, 38). The elongation processivity difference may be due to the length and sequence of the RNA template.

To further examine whether the RSV polymerase uses other primed RNAs as a template, we compared other terminal sequences of the RSV genome, such as Tr25, Le14, and LeC14, and supplied them with a short (5-nt) primer using [α-^32^P]GTP and NTPs. Interestingly, as shown in Fig. 5B, there were no differences between the template and primer-template complexes, such as Le14+p5̂ versus Le14 and LeC14+p5# versus LeC14. Next, we supplied only the incoming nucleotide for the polymerase reactions, as shown in Fig. 5C, and we observed the RNA elongation using the primer-template pair. As expected, neither p5̂ nor Le14 generated any products but Le14+p5̂ generated abortive products, as shown in Fig. 5C, lanes 1 to 3. LeC14 and Tr25 did not show a difference between the template with primer and the template without primer. Tr25 generated minor bands with and without primer, and this might have been due to the RSV polymerase activity on the formed secondary structure. Collectively, these results indicated that, besides *de novo* RNA synthesis, the RSV polymerase could catalyze primer-dependent template elongation.

### Sequence (position 1 to 5) specificity for RSV RNA synthesis.

**FIG 6:**
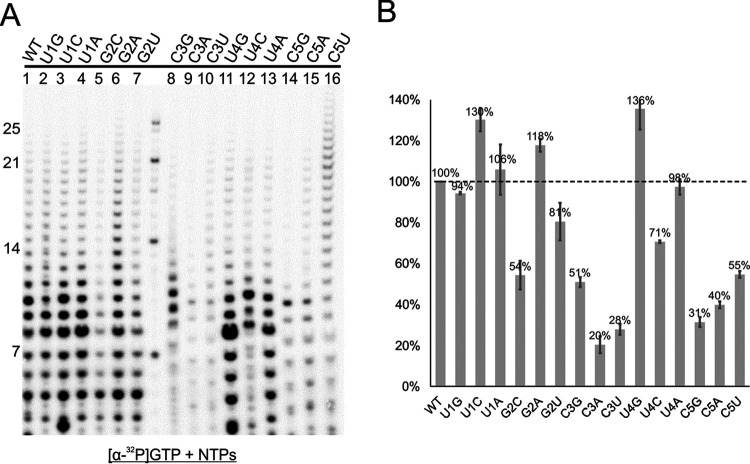
Sequence (positions 1 to 5) specificity for RSV RNA synthesis. (A) Polymerase activities of wt and single mutants of TrC10 using the RSV polymerase. The reaction mixtures were incubated in the presence of NTPs (ATP, CTP, and UTP each at 1.25 mM and GTP at 50 μM with 5 μCi of [α-^32^P]GTP). The middle lane shows the molecular weight ladder. The sequences of the RNA oligonucleotides are listed in Table 1. (B) Total polymerase activities of panel A were quantified and plotted. The sample size was *n* = 2, and the error bars represent the standard deviation. The dashed line indicates the polymerase activities of wt TrC10.

We surveyed a minimal length of RNA with the aforementioned experiments. We further tested whether the polymerase could initiate synthesis on a template with mutated sequences. The single mutations of positions 1, 2, 3, 4, and 5 were generated from the mini-template TrC10 that we identified (Table 1). The polymerase assay results are shown in Fig. 6A. The quantification of the polymerase activities is shown in Fig. 6B, where we plotted the polymerase activity of the wt template as 100%. As expected, many but not all mutations were detrimental and yielded reduced polymerase activities. No single mutation caused absolute abolishment of the polymerase activities, suggesting that the polymerase is tolerant to certain changes while retaining high specificity. Among mutations of the first five positions, positions 1, 2, and 4 were most tolerant of the mutations, and positions 3 and 5 were most sensitive to them (Fig. 6B). Mutations at positions 3 and 5 yielded 20% to 50% reduced activity, suggesting the importance of the sequences at those two positions. Interestingly, several mutations (U1C, U1A, G2A, and U4G) yielded increased polymerase activities, compared to the wt sequence. In particular, the U4G mutation switched the first five nucleotide sequences of the TrC template to those of the Le template (TrC, 3′-UGC**U**C; Le, 3′-UGC**G**C). U4G yielded higher activity than did the wt sequence, which suggests that the Le template is a slightly stronger promoter than that of the TrC template for polymerase activities at the length of 10 nt. Those results are consistent with previous reports (27).

### Effects of primer match or mismatch for RNA elongation.

**FIG 7:**
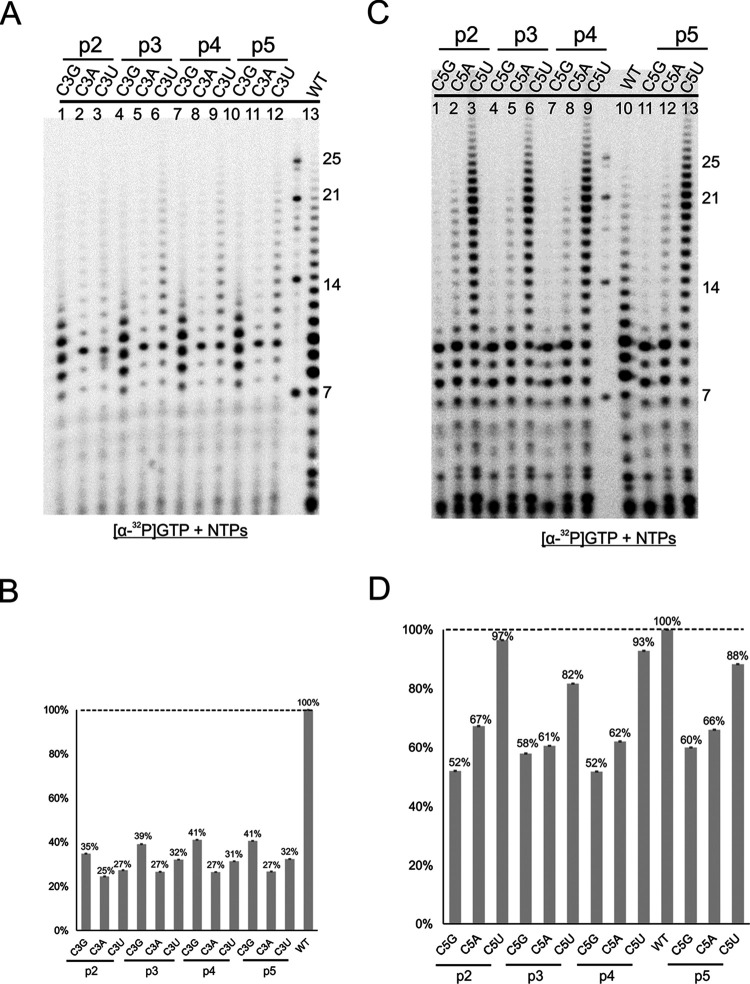
Position 3 and 5 mutations with primers 2 to 5. (A) Single mutants at position 3 of TrC10, C3G, C3A, and C3U were incubated with p2, p3, p4, and p5 for the RSV polymerase activities. The reaction mixtures were incubated in the presence of NTPs (ATP, CTP, and UTP each at 1.25 mM and GTP at 50 μM with 5 μCi of [α-^32^P]GTP). The right lane shows the molecular weight ladder. The sequences of the RNA oligonucleotides are listed in Table 1. (B) Total polymerase activities in panel A were quantified and plotted. The dashed line indicates the polymerase activities of wt TrC10. (C) Single mutants at position 5 of TrC10, C5G, C5A, and C5U were incubated with p2, p3, p4, and p5 for the RSV polymerase activities. The reaction mixtures were incubated in the presence of NTPs (ATP, CTP, and UTP each at 1.25 mM and GTP at 50 μM with 5 μCi of [α-^32^P]GTP). The right lane shows the molecular weight ladder. The sequences of the RNA oligonucleotides are listed in Table 1. (D) Total polymerase activities in panel C were quantified and plotted. The dashed line indicates the polymerase activities of wt TrC10.

We demonstrated that we could supply primers to RNA templates for RNA elongation using the RSV polymerase. We wanted to know whether we could see active elongation using mismatched primers. Because positions 3 and 5 had the most severe phenotypes, we examined the polymerase activities using mutations at both sites. For both positions, we used 2-, 3-, 4-, and 5-nt long primers (p2, p3, p4, and p5, respectively) to compare the polymerase activities (Table 1). As expected, all mutations at position 3 yielded reduced activities, ranging from 25% to 41% for all template-primer pairs, and there was no significant difference among primers with different lengths (Fig. 7A and B). Interestingly, those reduced rates were comparable to those without primers, at 20% to 51% (Fig. 6). For position 5, all mutations yielded less reduced activities with primers, at 52% to 97% (Fig. 7C and D), than without primers, at 31% to 55% (Fig. 6). There was no significant difference among the varied lengths of the primers used in the reactions for the same template.

### Sequence (position 6 to 12) specificity for RSV RNA synthesis.

**FIG 8:**
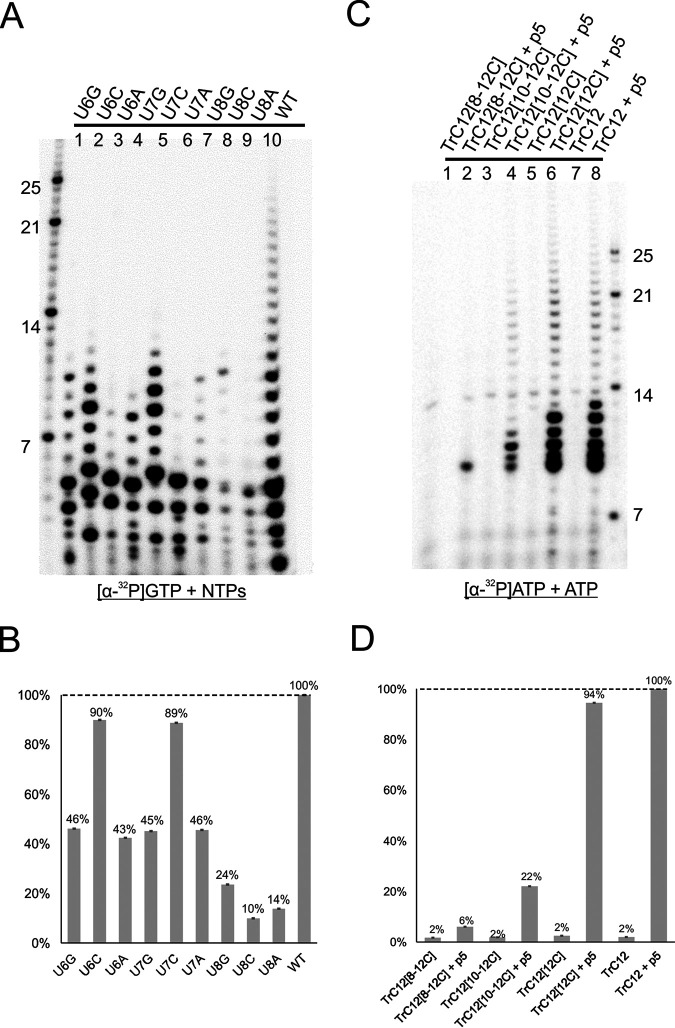
Sequence (positions 6 to 12) specificity for RSV RNA synthesis. (A) The polymerase activities of wt TrC10 and single mutants (positions 6 to 8) of TrC10 using the RSV polymerase were determined. The reaction mixtures were incubated in the presence of NTPs (ATP, CTP, and UTP each at 1.25 mM and GTP at 50 μM with 5 μCi of [α-^32^P]GTP). The left lane shows the molecular weight ladder. The sequences of the RNA oligonucleotides are listed in Table 1. (B) Total polymerase activities in panel A were quantified and plotted. The dashed line indicates the polymerase activities of wt TrC10. (C) The polymerase activities of wt and mutant TrC12 using the RSV polymerase were determined. The reaction mixtures were incubated in the presence of ATP (ATP at 50 μM with 5 μCi of [α-^32^P]ATP) (lanes 1 to 8). The right lane shows the molecular weight ladder. The sequences of the RNA oligonucleotides are listed in Table 1. (D) Total polymerase activities in panel C were quantified and plotted. The dashed line indicates the polymerase activities of wt TrC12.

To map the essential promoter sequence, we performed additional single mutations at positions 6, 7, and 8 based on TrC10 (sequences in Table 1). We used [α-^32^P]GTP and NTPs for the assay. The generated products are shown in Fig. 8A, and the quantification of the polymerase activities is shown in Fig. 8B. Most of the mutations led to a >50% loss of polymerase activity (Fig. 8B). Mutation at position 8 was the most severe among all three, with only 10% to 24% of polymerase activity, compared to the wt activity (Fig. 8A, lanes 7 to 9). Mutations at positions 6 and 7 caused about 50% reductions, except the U-to-C mutation, which retained about 90% of polymerase activity (Fig. 8A, lanes 2 and 5). This might be because changing U to C increased the use of GTP and, thus [α-^32^P]GTP, elevating our measured activity level. Interestingly, the U-to-C mutation at position 8 had the lowest (10%) activity. This result highlights the critical position of the promoter. Compared to positions 1 to 5, there was no enhancement of the polymerase activity, compared to the wt activity, suggesting those promoter sequences might be conserved through evolution.

To further elucidate the sequence specificity of the promoter, we swapped one or more Us to Cs from positions 6 to 12 (Table 1). We used [α-^32^P]ATP as the labeling nucleotide, and we compared the polymerase activity of the swap mutants with or without a p5 primer. Results showed that mutations at positions 8 to 12, 10 to 12, or 12 without a primer retained only a background 2% of polymerase activity (Fig. 8C and D). Interestingly, the polymerase activity with the mutations at positions 10 to 12 was restored about 10-fold using the primer (22% versus 2%), and position 12 could be compensated to nearly full activities (94% versus 2%). Because we supplied only [α-^32^P]ATP or ATP in the reaction mixtures, it was expected that only the templates with p5 would have polymerase activity. Again, all synthesized bands were larger than 9 nt, despite position 6 being the first nucleotide addition site (Fig. 8C and D).

## DISCUSSION

In summary, we successfully prepared highly pure, full-length, wt and mutant RSV polymerases. In addition, our work shows that the purified recombinant RSV polymerase carries out not only *de novo* initiation on the naked RNA templates but also primer-based elongation based on a primer-template. In both initiation and elongation, the RSV polymerase is not fully processive, and it was known that the full processivity of the polymerase requires N protein-encapsidated RNA templates (1, 2). We defined the minimal length of the RNA template for *de novo* RNA synthesis of the RSV polymerase as 8 nt, shorter than previously reported (33, 34, 38). We showed that the RSV polymerase catalyzes primer-dependent RNA elongation with different lengths of primers on both short (10-nt) and long (25-nt) RNA templates. We fine mapped the promoter sequence for RNA synthesis by mutagenesis of the RNA template and identified critical positions for the *in vitro* RSV polymerase activity, consistent with the results previously obtained with the *in vivo* minigenome assay (27). We also examined the effects of the primers on the elongation of the RNA mutant templates. Overall, these findings agreed well with previous biochemical studies and provided additional insights into the initiation and elongation of RSV RNA synthesis (25–42).

### Preparation of the RSV polymerase.

It was traditionally challenging to obtain sufficient amounts of high-quality polymerase for mechanistic studies, due to the large size and instability of L protein (a single-chain polypeptide of > 2,100 amino acids, i.e., ∼250 kDa). In VSV and RABV, the L proteins can be isolated independently from the P proteins (37, 48). In RSV, however, the L proteins need to be coexpressed and isolated together (34). Interestingly, in NiV and EBOV, the L proteins also need to be coexpressed with P or VP35 (and VP30) (38, 39). Among those viruses, VSV and RABV belong to the same family, *Rhabdoviridae*, while RSV, NiV, and EBOV belong to *Pneumoviridae*, *Paramyxoviridae*, and *Filoviridae*, respectively (56). The differences among the viruses may be due not only to different virus families but also to different interactions among L and its cofactors established in each family. The recombinant RSV L-P complex was made and functionally tested previously by Fearns and Deval and colleagues (33, 34, 40, 53). The samples usually contained additional contaminants. Given the overall low yield of the RSV polymerase, we optimized the strategy and protocol that was initially established by Fearns and colleagues (34), and we managed to obtain a large quantity of highly pure and active RSV polymerase. Compared to the His tag on the RSV P (34), we found that use of the 6×His-TEV tag on the RSV L with additional TEV protease cleavage steps, followed by Ni-NTA purification, significantly increased the purity of the L-P complex. The quality of our purified RSV polymerases is comparable to that in another recent cryo-EM structural study in which a slightly different strategy was used, involving the RSV L with an N-terminal dual Strep-tag and the RSV P with a C-terminal 6×His tag (49). Regarding the negative-stain EM images of the potential oligomeric states of the polymerase, we could not distinguish whether those are functional oligomers of the RSV L-P complex; further analysis is needed to rule this out. Collectively, this revised protocol supplied high-quality protein samples and set the stage for further mechanistic analyses of RSV RNA synthesis.

### *De novo* initiation of RNA synthesis.

The *in vitro* reconstitution of *de novo* NNS RNA synthesis was successfully studied in RSV and several other systems (34–39, 57–60). As highlighted above, in RSV, EBOV, and NiV, L and P need to be coexpressed and copurified. In VSV and RABV, however, L and P can be isolated separately. Therefore, there are strategic differences for *in vitro* assays. For VSV and RABV, the enzymatic activities of L can be examined in the presence and absence of P but not in many other systems because of the coexistence of L and P during preparation. This may also be related to the specificities and differences of different virus families. For example, previous studies showed that VSV L and RABV L initiate RNA synthesis only at the 3′ end (+1) but RSV L initiates *de novo* RNA synthesis at both the 3′ end (+1) and internal (+3) positions (34, 36, 37). It was also shown that the template RNA was able to fold into a secondary structure for the RSV polymerase to extend at the 3′ end using a back-priming mechanism (34), but that had not been reported for many other viruses. In this study, we were able to reproduce the RNA synthesis activities using a 25-nt TrC RNA template (36). We also compared much shorter RNA templates, such as ≤14 nt. We defined the minimal length of RNA that serves as a template for the RSV polymerase to be 8 nt. As we showed in this study, we found that positions 3, 5, and 8 of the promoter sequence are vital for the polymerase activity. It is possible that RNA oligonucleotides shorter than 8 nt do not have all promoter elements necessary for RNA synthesis. Therefore, the minimal length of the promoter sequence for the RSV polymerase is 8 nt.

### Primer-based elongation using the RSV polymerase.

RdRPs are the catalytic core of RNA synthesis and the key players in the life cycle of RNA viruses. Proper initiation is critical to ensure the integrity of the viral RNA genome. There are two main mechanisms by which viral RNA synthesis can be initiated, i.e., primer independent (or *de novo*) and primer dependent (see reviews in references 61–64). Briefly, for *de novo* initiation, the initiating nucleotide supplies the 3′-OH for addition of the second nucleotide, whereas primer-dependent initiation requires the use of either an oligonucleotide or a protein primer to provide the 3′-OH nucleophile for extension (elongation). Like the primer-dependent elongation in EBOV and NiV (38, 39), we demonstrated that the RSV polymerase carries out not only *de novo* RNA synthesis but also primer-dependent elongation. We supplied short RNA oligonucleotides as primers (2 nt to 5 nt) to pair with the 5′ end of the RNA templates to examine the RNA elongation. Then we added the incoming nucleotide to the preannealed primer-template for RNA synthesis. This study used longer wt templates (25 nt and 14 nt), compared to the shorter mutant template (11 nt) reported previously (40), and the primer-dependent elongation showed a slightly different pattern, with the first predominant product at 9 nt. This is likely due to the longer length of the template allowing further extension of the product and more processivity at the initial elongation stage. An alternative explanation is that the chemical nature of the primers influenced such migration patterns.

### Fine mapping of the promoter sequence of the RSV polymerase.

We identified the minimal length template for RNA synthesis as 8 nt, which paved the way for further examining the template with swapped sequences. To map the essential promoter sequence, we performed single mutations at positions 1 through 8. Our work suggests that the RSV polymerase initiates *de novo* RNA synthesis by recognizing the promoter sequence, rather than a single nucleotide at the 3′ end of the templates. Together, we identified positions 3, 5, and 8 to be the most critical positions for polymerase activity. Our *in vitro* mapping of the promoter sequence agreed well with the previous study showing that positions 3, 5, and 8 of the promoter sequence are critical, using a minigenome assay, by Collins and Fearns and colleagues (27, 30).

Together, the purified RSV polymerase and established assays represent a robust system for delineating the enzymatic function of the RSV polymerase. The knowledge gained from this system readily adds to the pool of knowledge regarding RSV RNA synthesis. The findings described here revealed the complexity of RSV RNA synthesis. In particular, the RSV polymerase could carry out both *de novo* and primer-dependent RNA synthesis, similar to several other RNA viruses. Our mapping of the critical residues could be helpful in identifying a suitable RNA template for further in-depth functional and structural analyses. One limitation of the *in vitro* assay system, like many other systems in the field, lacks N protein-encapsidated RNA templates. Thus, one future direction will be to establish the N-RNA templates for a better understanding of the RSV RNA synthesis.

## MATERIALS AND METHODS

### Expression and purification of the RSV polymerase (L-P complex).

The helper plasmids of codon-optimized sequences of the RSV (strain A2) L and P proteins were provided as a generous gift from Martin Moore (Emory). The L and P genes were subcloned into the pFastBac Dual vector (Invitrogen) with the RSV L gene at open reading frame 1 (ORF1) and the RSV P gene at ORF2. A 6×His tag was added to the N terminus of the RSV L protein, separated by a TEV protease cleavage site. The RSV L(D811A)-P mutant was generated using PCR-based site-directed mutagenesis, with the plasmid encoding the wt RSV L-P complex as the template. Then, the recombinant pFastBac Dual vector was transformed into Escherichia coli DH10Bac for bacmid DNA generation. The Cellfectin II reagent (Thermo Fisher Scientific) was used to transfect the bacmid DNA into Sf21 cells to obtain the recombinant baculoviruses. Sf21 cells were infected by the recombinant baculoviruses in suspension culture and harvested 72 h postinfection by centrifugation for 15 min at 1,000 × *g*. Cells were resuspended in lysis buffer (50 mM sodium phosphate [pH 7.4], 300 mM NaCl, 6 mM MgSO_4_, 10% glycerol, 0.2% NP-40, EDTA-free protease inhibitor), lysed with an homogenizer, and clarified through centrifugation for 60 min at 16,000 × *g*. The clarified lysate was incubated with Co^2+^-NTA agarose resin (GoldBio) and washed with wash buffer (50 mM sodium phosphate [pH 7.4], 300 mM NaCl, 6 mM MgSO_4_, 10% glycerol, 10 mM imidazole), and the RSV L-P complexes were eluted with elution buffer (50 mM sodium phosphate [pH 7.4], 300 mM NaCl, 6 mM MgSO_4_, 10% glycerol, 250 mM imidazole). The eluted sample was then treated with TEV enzyme and applied to Co^2+^-NTA agarose resin again. The flowthrough sample was applied to a heparin column and further purified by size exclusion chromatography with gel filtration buffer [25 mM HEPES [pH 7.4], 300 mM NaCl, 6 mM MgSO_4_, 0.5 mM tris(2-carboxyethyl)phosphine hydrochloride [TCEP]] using a Superose 6 Increase 10/300 GL column (GE Healthcare). SDS-PAGE analyzed the quality of purified proteins. The bands migrating at ∼250 kDa and ∼35 kDa were confirmed to be the RSV L and P polypeptides by liquid chromatography-mass spectrometry (45). The pure proteins were flash-frozen in liquid nitrogen and stored in 30-μl aliquots at −80 °C for further use. The mutant L(D811A)-P complex was expressed, purified, and stored in the same manner as the wt L-P complex.

### *In vitro* RNA synthesis assay.

The terminal sequences of the genome or antigenome, such as the Le and TrC promoter sequences, were used in the RNA synthesis assay. All RNA oligonucleotides were chemically synthesized by IDT or Dharmacon. Radioactive isotope-labeled nucleotides [α-^32^P]GTP, [α-^32^P]ATP, [α-^32^P]UTP, and [γ-^32^P]ATP were purchased from Perkin Elmer. The reaction mixtures contained 2 μM RNA template (without or with 2 μM primer), the RSV L-P complexes (∼300 ng RSV L), NTPs (ATP, CTP, and UTP each at 1.25 mM and GTP at 50 μM with 5 μCi of [α-^32^P]GTP) (figure legends indicate the details for each reaction mixture), and reaction buffer (50 mM Tris-HCl [pH 7.4], 8 mM MgCl_2_, 5 mM dithiothreitol, 10% glycerol) in a final volume of 20 μl. The reaction mixtures were incubated at 30°C for 3 h and heated to 90°C for 3 min, and then 5 μl of the stop buffer (90% formamide, 20 mM EDTA, 0.02% bromophenol blue) was added to each reaction mixture. Other radioactive isotope-labeled nucleotides ([α-^32^P]ATP, [α-^32^P]UTP, and [γ-^32^P]ATP) were incubated similarly as [α-^32^P]GTP (figure legends indicate the details for each reaction mixture). The isotope-labeled nucleotides with the same concentration were freshly purchased and used for the reactions. For clarity, we directly compared only the reaction mixtures containing the same radioactive isotope-labeled NTPs. The RNA products were analyzed by electrophoresis on a 20% polyacrylamide gel containing 7 M urea in a Tris-borate-EDTA buffer, followed by phosphorimaging with a Typhoon FLA 7000 scanner (GE Healthcare). The quantification of the images was carried out with an analysis toolbox from ImageQuant TL software (GE Healthcare). We analyzed the images using area- and profile-based tools and selected the corresponding area of each lane with a box for calculation by the software. The molecular weight ladders were generated by labeling Tr7, Tr14, Tr21, and Tr25 with [γ-^32^P]ATP using polynucleotide kinase and following the protocols according to the manufacturer (NEB).

### DLS experiments.

DLS experiments were performed on a DynaPro plate reader II (Wyatt Technologies). Measurements of the RSV polymerase (L-P complex) samples (1 mg/ml) were obtained in gel filtration buffer at 25°C and analyzed using Dynamics software (Wyatt).

### Negative-stain EM.

Samples were prepared on continuous carbon films supported by 400-mesh copper grids (Ted Pella). A 3-μl drop of the RSV L-P was applied to a freshly glow-discharged grid, blotted to a thin film with filter paper, and immediately stained with 1% (wt/vol) uranyl formate. EM was performed using an FEI Talos L120C electron microscope, operating at 120 keV, equipped with an FEI Ceta 4,000 × 4,000-pixel charge-coupled device (CCD) camera. Images were collected at nominal magnifications of ×73,000 (1.97 Å/pixel). The images were acquired at defocus values of −1.2 to −2.0 μm and electron doses of ∼25 e^−^/Å^2^.

## Supplementary Material

Supplemental file 1

## References

[1] Collins PL, Fearns R, Graham BS 2013 Respiratory syncytial virus: virology, reverse genetics, and pathogenesis of disease. Curr Top Microbiol Immunol 372:3–38. doi:10.1007/978-3-642-38919-1_1. 24362682 PMC4794264

[2] Collins PL, Karron RA 2013 Respiratory syncytial virus and metapneumovirus, p 1087–1118. *In* Knipe DM, Howley PM, Griffin DE, Lamb RA, Martin MA, Roizman B, Straus SE (ed), Fields virology, 6th ed vol 1 Lippincott Williams & Wilkins, Philadelphia, PA.

[3] Nair H, Nokes DJ, Gessner BD, Dherani M, Madhi SA, Singleton RJ, O'Brien KL, Roca A, Wright PF, Bruce N, Chandran A, Theodoratou E, Sutanto A, Sedyaningsih ER, Ngama M, Munywoki PK, Kartasasmita C, Simoes EA, Rudan I, Weber MW, Campbell H 2010 Global burden of acute lower respiratory infections due to respiratory syncytial virus in young children: a systematic review and meta-analysis. Lancet 375:1545–1555. doi:10.1016/S0140-6736(10)60206-1. 20399493 PMC2864404

[4] Neuzil KM 2016 Progress toward a respiratory syncytial virus vaccine. Clin Vaccine Immunol 23:186–188. doi:10.1128/CVI.00037-16. 26818954 PMC4783429

[5] Jorquera PA, Tripp RA 2017 Respiratory syncytial virus: prospects for new and emerging therapeutics. Expert Rev Respir Med 11:609–615. doi:10.1080/17476348.2017.1338567. 28574729

[6] Shi T, McAllister DA, O'Brien KL, Simoes EAF, Madhi SA, Gessner BD, Polack FP, Balsells E, Acacio S, Aguayo C, Alassani I, Ali A, Antonio M, Awasthi S, Awori JO, Azziz-Baumgartner E, Baggett HC, Baillie VL, Balmaseda A, Barahona A, Basnet S, Bassat Q, Basualdo W, Bigogo G, Bont L, Breiman RF, Brooks WA, Broor S, Bruce N, Bruden D, Buchy P, Campbell S, Carosone-Link P, Chadha M, Chipeta J, Chou M, Clara W, Cohen C, de Cuellar E, Dang DA, Dash-Yandag B, Deloria-Knoll M, Dherani M, Eap T, Ebruke BE, Echavarria M, de Freitas Lazaro Emediato CC, Fasce RA, Feikin DR, Feng L, Gentile A, Gordon A, Goswami D, Goyet S, Groome M, Halasa N, Hirve S, Homaira N, Howie SRC, Jara J, Jroundi I, Kartasasmita CB, Khuri-Bulos N, Kotloff KL, Krishnan A, Libster R, Lopez O, Lucero MG, Lucion F, Lupisan SP, Marcone DN, McCracken JP, Mejia M, Moisi JC, Montgomery JM, Moore DP, Moraleda C, Moyes J, Munywoki P, Mutyara K, Nicol MP, Nokes DJ, Nymadawa P, da Costa Oliveira MT, Oshitani H, Pandey N, Paranhos-Baccalà G, Phillips LN, Picot VS, Rahman M, Rakoto-Andrianarivelo M, Rasmussen ZA, Rath BA, Robinson A, Romero C, Russomando G, Salimi V, Sawatwong P, Scheltema N, Schweiger B, Scott JAG, Seidenberg P, Shen K, Singleton R, Sotomayor V, Strand TA, Sutanto A, Sylla M, Tapia MD, Thamthitiwat S, Thomas ED, Tokarz R, Turner C, Venter M, Waicharoen S, Wang J, Watthanaworawit W, Yoshida LM, Yu H, Zar HJ, Campbell H, Nair H 2017 Global, regional, and national disease burden estimates of acute lower respiratory infections due to respiratory syncytial virus in young children in 2015: a systematic review and modelling study. Lancet 390:946–958. doi:10.1016/S0140-6736(17)30938-8. 28689664 PMC5592248

[7] Whelan SP, Barr JN, Wertz GW 2004 Transcription and replication of nonsegmented negative-strand RNA viruses. Curr Top Microbiol Immunol 283:61–119. doi:10.1007/978-3-662-06099-5_3. 15298168

[8] Conzelmann KK 1998 Nonsegmented negative-strand RNA viruses: genetics and manipulation of viral genomes. Annu Rev Genet 32:123–162. doi:10.1146/annurev.genet.32.1.123. 9928477

[9] Poch O, Sauvaget I, Delarue M, Tordo N 1989 Identification of four conserved motifs among the RNA-dependent polymerase encoding elements. EMBO J 8:3867–3874. doi:10.1002/j.1460-2075.1989.tb08565.x. 2555175 PMC402075

[10] Poch O, Blumberg BM, Bougueleret L, Tordo N 1990 Sequence comparison of five polymerases (L proteins) of unsegmented negative-strand RNA viruses: theoretical assignment of functional domains. J Gen Virol 71:1153–1162. doi:10.1099/0022-1317-71-5-1153. 2161049

[11] Stec DS, Hill MG III, Collins PL 1991 Sequence analysis of the polymerase L gene of human respiratory syncytial virus and predicted phylogeny of nonsegmented negative-strand viruses. Virology 183:273–287. doi:10.1016/0042-6822(91)90140-7. 2053282

[12] Mazumder B, Barik S 1994 Requirement of casein kinase II-mediated phosphorylation for the transcriptional activity of human respiratory syncytial viral phosphoprotein P: transdominant negative phenotype of phosphorylation-defective P mutants. Virology 205:104–111. doi:10.1006/viro.1994.1624. 7975205

[13] Grosfeld H, Hill MG, Collins PL 1995 RNA replication by respiratory syncytial virus (RSV) is directed by the N, P, and L proteins; transcription also occurs under these conditions but requires RSV superinfection for efficient synthesis of full-length mRNA. J Virol 69:5677–5686. doi:10.1128/JVI.69.9.5677-5686.1995. 7637014 PMC189426

[14] Marriott AC, Wilson SD, Randhawa JS, Easton AJ 1999 A single amino acid substitution in the phosphoprotein of respiratory syncytial virus confers thermosensitivity in a reconstituted RNA polymerase system. J Virol 73:5162–5165. doi:10.1128/JVI.73.6.5162-5165.1999. 10233981 PMC112563

[15] Cowton VM, McGivern DR, Fearns R 2006 Unravelling the complexities of respiratory syncytial virus RNA synthesis. J Gen Virol 87:1805–1821. doi:10.1099/vir.0.81786-0. 16760383

[16] Munday DC, Wu W, Smith N, Fix J, Noton SL, Galloux M, Touzelet O, Armstrong SD, Dawson JM, Aljabr W, Easton AJ, Rameix-Welti MA, de Oliveira AP, Simabuco FM, Ventura AM, Hughes DJ, Barr JN, Fearns R, Digard P, Eleouet JF, Hiscox JA 2015 Interactome analysis of the human respiratory syncytial virus RNA polymerase complex identifies protein chaperones as important cofactors that promote L-protein stability and RNA synthesis. J Virol 89:917–930. doi:10.1128/JVI.01783-14. 25355874 PMC4300676

[17] Fearns R, Plemper RK 2017 Polymerases of paramyxoviruses and pneumoviruses. Virus Res 234:87–102. doi:10.1016/j.virusres.2017.01.008. 28104450 PMC5476513

[18] Noton SL, Fearns R 2015 Initiation and regulation of paramyxovirus transcription and replication. Virology 479–480:545–554. doi:10.1016/j.virol.2015.01.014. PMC4424093 25683441

[19] Tawar RG, Duquerroy S, Vonrhein C, Varela PF, Damier-Piolle L, Castagne N, MacLellan K, Bedouelle H, Bricogne G, Bhella D, Eleouet JF, Rey FA 2009 Crystal structure of a nucleocapsid-like nucleoprotein-RNA complex of respiratory syncytial virus. Science 326:1279–1283. doi:10.1126/science.1177634. 19965480

[20] Abraham G, Banerjee AK 1976 Sequential transcription of the genes of vesicular stomatitis virus. Proc Natl Acad Sci U S A 73:1504–1508. doi:10.1073/pnas.73.5.1504. 179088 PMC430325

[21] Ball LA, White CN 1976 Order of transcription of genes of vesicular stomatitis virus. Proc Natl Acad Sci U S A 73:442–446. doi:10.1073/pnas.73.2.442. 174107 PMC335925

[22] Piedra FA, Qiu X, Teng MN, Avadhanula V, Machado AA, Kim DK, Hixson J, Bahl J, Piedra PA 2020 Non-gradient and genotype-dependent patterns of RSV gene expression. PLoS One 15:e0227558. doi:10.1371/journal.pone.0227558. 31923213 PMC6953876

[23] Pagan I, Holmes EC, Simon-Loriere E 2012 Level of gene expression is a major determinant of protein evolution in the viral order *Mononegavirales*. J Virol 86:5253–5263. doi:10.1128/JVI.06050-11. 22345453 PMC3347393

[24] Dickens LE, Collins PL, Wertz GW 1984 Transcriptional mapping of human respiratory syncytial virus. J Virol 52:364–369. doi:10.1128/JVI.52.2.364-369.1984. 6492254 PMC254535

[25] Kuo L, Grosfeld H, Cristina J, Hill MG, Collins PL 1996 Effects of mutations in the gene-start and gene-end sequence motifs on transcription of monocistronic and dicistronic minigenomes of respiratory syncytial virus. J Virol 70:6892–6901. doi:10.1128/JVI.70.10.6892-6901.1996. 8794332 PMC190738

[26] Fearns R, Collins PL, Peeples ME 2000 Functional analysis of the genomic and antigenomic promoters of human respiratory syncytial virus. J Virol 74:6006–6014. doi:10.1128/jvi.74.13.6006-6014.2000. 10846082 PMC112097

[27] Fearns R, Peeples ME, Collins PL 2002 Mapping the transcription and replication promoters of respiratory syncytial virus. J Virol 76:1663–1672. doi:10.1128/jvi.76.4.1663-1672.2002. 11799161 PMC135899

[28] Peeples ME, Collins PL 2000 Mutations in the 5′ trailer region of a respiratory syncytial virus minigenome which limit RNA replication to one step. J Virol 74:146–155. doi:10.1128/jvi.74.1.146-155.2000. 10590101 PMC111523

[29] Cowton VM, Fearns R 2005 Evidence that the respiratory syncytial virus polymerase is recruited to nucleotides 1 to 11 at the 3′ end of the nucleocapsid and can scan to access internal signals. J Virol 79:11311–11322. doi:10.1128/JVI.79.17.11311-11322.2005. 16103183 PMC1193587

[30] Noton SL, Cowton VM, Zack CR, McGivern DR, Fearns R 2010 Evidence that the polymerase of respiratory syncytial virus initiates RNA replication in a nontemplated fashion. Proc Natl Acad Sci U S A 107:10226–10231. doi:10.1073/pnas.0913065107. 20479224 PMC2890450

[31] Noton SL, Fearns R 2011 The first two nucleotides of the respiratory syncytial virus antigenome RNA replication product can be selected independently of the promoter terminus. RNA 17:1895–1906. doi:10.1261/rna.2813411. 21878549 PMC3185921

[32] Noton SL, Aljabr W, Hiscox JA, Matthews DA, Fearns R 2014 Factors affecting de novo RNA synthesis and back-priming by the respiratory syncytial virus polymerase. Virology 462–463:318–327. doi:10.1016/j.virol.2014.05.032. PMC4125506 25010481

[33] Deval J, Hong J, Wang G, Taylor J, Smith LK, Fung A, Stevens SK, Liu H, Jin Z, Dyatkina N, Prhavc M, Stoycheva AD, Serebryany V, Liu J, Smith DB, Tam Y, Zhang Q, Moore ML, Fearns R, Chanda SM, Blatt LM, Symons JA, Beigelman L 2015 Molecular basis for the selective inhibition of respiratory syncytial virus RNA polymerase by 2′-fluoro-4′-chloromethyl-cytidine triphosphate. PLoS Pathog 11:e1004995. doi:10.1371/journal.ppat.1004995. 26098424 PMC4476725

[34] Noton SL, Deflube LR, Tremaglio CZ, Fearns R 2012 The respiratory syncytial virus polymerase has multiple RNA synthesis activities at the promoter. PLoS Pathog 8:e1002980. doi:10.1371/journal.ppat.1002980. 23093940 PMC3475672

[35] Tremaglio CZ, Noton SL, Deflube LR, Fearns R 2013 Respiratory syncytial virus polymerase can initiate transcription from position 3 of the leader promoter. J Virol 87:3196–3207. doi:10.1128/JVI.02862-12. 23283954 PMC3592119

[36] Morin B, Rahmeh AA, Whelan SP 2012 Mechanism of RNA synthesis initiation by the vesicular stomatitis virus polymerase. EMBO J 31:1320–1329. doi:10.1038/emboj.2011.483. 22246179 PMC3297992

[37] Morin B, Liang B, Gardner E, Ross RA, Whelan SPJ 2017 An in vitro RNA synthesis assay for rabies virus defines ribonucleoprotein interactions critical for polymerase activity. J Virol 91:e01508-16. doi:10.1128/JVI.01508-16. 27795419 PMC5165209

[38] Tchesnokov EP, Raeisimakiani P, Ngure M, Marchant D, Gotte M 2018 Recombinant RNA-dependent RNA polymerase complex of Ebola virus. Sci Rep 8:3970. doi:10.1038/s41598-018-22328-3. 29507309 PMC5838098

[39] Jordan PC, Liu C, Raynaud P, Lo MK, Spiropoulou CF, Symons JA, Beigelman L, Deval J 2018 Initiation, extension, and termination of RNA synthesis by a paramyxovirus polymerase. PLoS Pathog 14:e1006889. doi:10.1371/journal.ppat.1006889. 29425244 PMC5823471

[40] Deval J, Fung A, Stevens SK, Jordan PC, Gromova T, Taylor JS, Hong J, Meng J, Wang G, Dyatkina N, Prhavc M, Symons JA, Beigelman L 2016 Biochemical effect of resistance mutations against synergistic inhibitors of RSV RNA polymerase. PLoS One 11:e0154097. doi:10.1371/journal.pone.0154097. 27163448 PMC4862670

[41] Ogino T, Green TJ 2019 RNA synthesis and capping by non-segmented negative strand RNA viral polymerases: lessons from a prototypic virus. Front Microbiol 10:1490. doi:10.3389/fmicb.2019.01490. 31354644 PMC6636387

[42] Ogino T, Banerjee AK 2007 Unconventional mechanism of mRNA capping by the RNA-dependent RNA polymerase of vesicular stomatitis virus. Mol Cell 25:85–97. doi:10.1016/j.molcel.2006.11.013. 17218273

[43] Llorente MT, Taylor IA, Lopez-Vinas E, Gomez-Puertas P, Calder LJ, Garcia-Barreno B, Melero JA 2008 Structural properties of the human respiratory syncytial virus P protein: evidence for an elongated homotetrameric molecule that is the smallest orthologue within the family of paramyxovirus polymerase cofactors. Proteins 72:946–958. doi:10.1002/prot.21988. 18300250

[44] Brown G, Rixon HW, Steel J, McDonald TP, Pitt AR, Graham S, Sugrue RJ 2005 Evidence for an association between heat shock protein 70 and the respiratory syncytial virus polymerase complex within lipid-raft membranes during virus infection. Virology 338:69–80. doi:10.1016/j.virol.2005.05.004. 15936795

[45] Cao D, Gao Y, Roesler C, Rice S, D'Cunha P, Zhuang L, Slack J, Domke M, Antonova A, Romanelli S, Keating S, Forero G, Juneja P, Liang B 2020 Cryo-EM structure of the respiratory syncytial virus RNA polymerase. Nat Commun 11:368. doi:10.1038/s41467-019-14246-3. 31953395 PMC6969064

[46] Castagne N, Barbier A, Bernard J, Rezaei H, Huet JC, Henry C, Costa BD, Eleouet JF 2004 Biochemical characterization of the respiratory syncytial virus P-P and P-N protein complexes and localization of the P protein oligomerization domain. J Gen Virol 85:1643–1653. doi:10.1099/vir.0.79830-0. 15166449

[47] Sourimant J, Rameix-Welti MA, Gaillard AL, Chevret D, Galloux M, Gault E, Eleouet JF 2015 Fine mapping and characterization of the L-polymerase-binding domain of the respiratory syncytial virus phosphoprotein. J Virol 89:4421–4433. doi:10.1128/JVI.03619-14. 25653447 PMC4442346

[48] Rahmeh AA, Schenk AD, Danek EI, Kranzusch PJ, Liang B, Walz T, Whelan SP 2010 Molecular architecture of the vesicular stomatitis virus RNA polymerase. Proc Natl Acad Sci U S A 107:20075–20080. doi:10.1073/pnas.1013559107. 21041632 PMC2993402

[49] Gilman MSA, Liu C, Fung A, Behera I, Jordan P, Rigaux P, Ysebaert N, Tcherniuk S, Sourimant J, Eleouet JF, Sutto-Ortiz P, Decroly E, Roymans D, Jin Z, McLellan JS 2019 Structure of the respiratory syncytial virus polymerase complex. Cell 179:193–204.e14. doi:10.1016/j.cell.2019.08.014. 31495574 PMC7111336

[50] Rahmeh AA, Morin B, Schenk AD, Liang B, Heinrich BS, Brusic V, Walz T, Whelan SP 2012 Critical phosphoprotein elements that regulate polymerase architecture and function in vesicular stomatitis virus. Proc Natl Acad Sci U S A 109:14628–14633. doi:10.1073/pnas.1209147109. 22908284 PMC3437890

[51] Zuker M 2003 Mfold web server for nucleic acid folding and hybridization prediction. Nucleic Acids Res 31:3406–3415. doi:10.1093/nar/gkg595. 12824337 PMC169194

[52] Noton SL, Nagendra K, Dunn EF, Mawhorter ME, Yu Q, Fearns R 2015 Respiratory syncytial virus inhibitor AZ-27 differentially inhibits different polymerase activities at the promoter. J Virol 89:7786–7798. doi:10.1128/JVI.00530-15. 25995255 PMC4505683

[53] Cressey TN, Noton SL, Nagendra K, Braun MR, Fearns R 2018 Mechanism for de novo initiation at two sites in the respiratory syncytial virus promoter. Nucleic Acids Res 46:6785–6796. doi:10.1093/nar/gky480. 29873775 PMC6061868

[54] Tchesnokov EP, Feng JY, Porter DP, Gotte M 2019 Mechanism of inhibition of Ebola virus RNA-dependent RNA polymerase by remdesivir. Viruses 11:326. doi:10.3390/v11040326. PMC6520719 30987343

[55] Ludeke B, Fearns R 2020 The respiratory syncytial virus polymerase can perform RNA synthesis with modified primers and nucleotide analogs. Virology 540:66–74. doi:10.1016/j.virol.2019.11.002. 31739186 PMC7737601

[56] Afonso CL, Amarasinghe GK, Bányai K, Bào Y, Basler CF, Bavari S, Bejerman N, Blasdell KR, Briand F-X, Briese T, Bukreyev A, Calisher CH, Chandran K, Chéng J, Clawson AN, Collins PL, Dietzgen RG, Dolnik O, Domier LL, Dürrwald R, Dye JM, Easton AJ, Ebihara H, Farkas SL, Freitas-Astúa J, Formenty P, Fouchier RAM, Fù Y, Ghedin E, Goodin MM, Hewson R, Horie M, Hyndman TH, Jiāng D, Kitajima EW, Kobinger GP, Kondo H, Kurath G, Lamb RA, Lenardon S, Leroy EM, Li C-X, Lin X-D, Liú L, Longdon B, Marton S, Maisner A, Mühlberger E, Netesov SV, Nowotny N, Patterson JL, Payne SL, Paweska JT, Randall RE, Rima BK, Rota P, Rubbenstroth D, Schwemmle M, Shi M, Smither SJ, Stenglein MD, Stone DM, Takada A, Terregino C, Tesh RB, Tian JH, Tomonaga K, Tordo N, Towner JS, Vasilakis N, Verbeek M, Volchkov VE, Wahl-Jensen V, Walsh JA, Walker PJ, Wang D, Wang LF, Wetzel T, Whitfield AE, Xiè JT, Yuen KY, Zhang YZ, Kuhn JH 2016 Taxonomy of the order Mononegavirales: update 2016. Arch Virol 161:2351–2360. doi:10.1007/s00705-016-2880-1. 27216929 PMC4947412

[57] Li J, Fontaine-Rodriguez EC, Whelan SP 2005 Amino acid residues within conserved domain VI of the vesicular stomatitis virus large polymerase protein essential for mRNA cap methyltransferase activity. J Virol 79:13373–13384. doi:10.1128/JVI.79.21.13373-13384.2005. 16227259 PMC1262600

[58] Li J, Wang JT, Whelan SP 2006 A unique strategy for mRNA cap methylation used by vesicular stomatitis virus. Proc Natl Acad Sci U S A 103:8493–8498. doi:10.1073/pnas.0509821103. 16709677 PMC1482520

[59] Li J, Rahmeh A, Morelli M, Whelan SP 2008 A conserved motif in region V of the large polymerase proteins of nonsegmented negative-sense RNA viruses that is essential for mRNA capping. J Virol 82:775–784. doi:10.1128/JVI.02107-07. 18003731 PMC2224588

[60] Rahmeh AA, Li J, Kranzusch PJ, Whelan SP 2009 Ribose 2′-*O* methylation of the vesicular stomatitis virus mRNA cap precedes and facilitates subsequent guanine-*N*-7 methylation by the large polymerase protein. J Virol 83:11043–11050. doi:10.1128/JVI.01426-09. 19710136 PMC2772757

[61] Ferrer-Orta C, Arias A, Escarmis C, Verdaguer N 2006 A comparison of viral RNA-dependent RNA polymerases. Curr Opin Struct Biol 16:27–34. doi:10.1016/j.sbi.2005.12.002. 16364629

[62] Ferrero D, Ferrer-Orta C, Verdaguer N 2018 Viral RNA-dependent RNA polymerases: a structural overview. Subcell Biochem 88:39–71. doi:10.1007/978-981-10-8456-0_3. 29900492

[63] Venkataraman S, Prasad B, Selvarajan R 2018 RNA dependent RNA polymerases: insights from structure, function and evolution. Viruses 10:76. doi:10.3390/v10020076. PMC5850383 29439438

[64] Te Velthuis AJ 2014 Common and unique features of viral RNA-dependent polymerases. Cell Mol Life Sci 71:4403–4420. doi:10.1007/s00018-014-1695-z. 25080879 PMC4207942

