# Supplementary material for: In Vitro Primer-Based RNA Elongation and Promoter Fine Mapping of the Respiratory Syncytial Virus
Source: J Virol. 2020 Dec 9;95(1):e01897-20. doi: 10.1128/JVI.01897-20 (PMC7737744; doi:10.1128/JVI.01897-20)
Supplement: Supplemental file 1 [file JVI.01897-20-s0001.pdf]

# *In vitro* primer-based RNA elongation and promoter fine mapping of the respiratory syncytial virus

## Supplementary Information

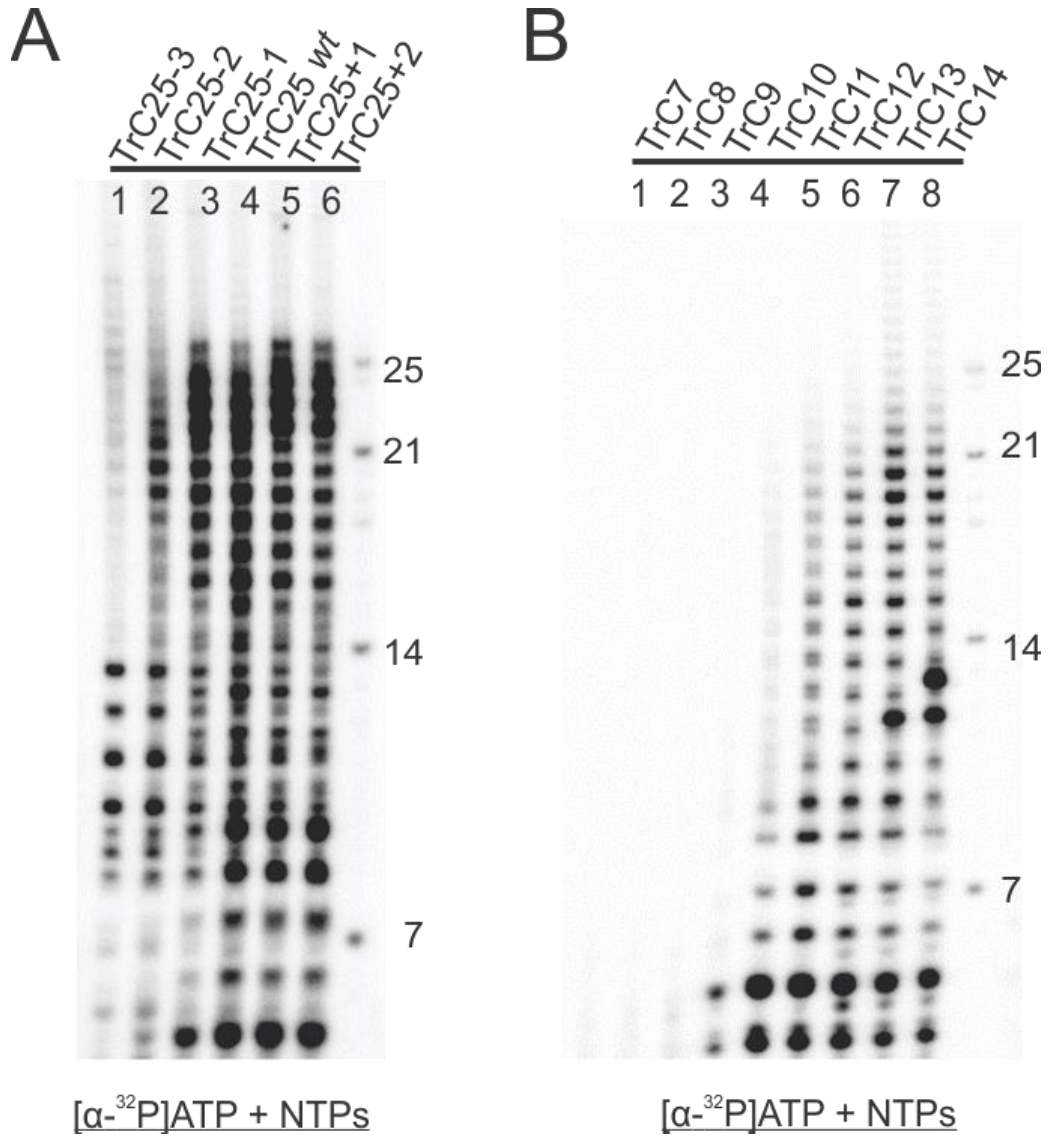


**Figure S1**: **Template length requirements for RNA synthesis**

(A) TrC25 and variants, as Figure 3A, were examined in the presence of NTPs (GTP, CTP, and UTP each at 1.25 mM and ATP at 50 μM with 5 μCi of [α-$^{32}$P]ATP) (lane 1-6). The deletion and addition of the 3' end of the RNA template based on TrC25, namely TrC25-3, TrC25-2, TrC25-1, TrC25+1, and TrC25+2 were used for examination of the template specificity. The right lane shows the molecular weight ladder. The sequences of the RNA oligos are listed in **Table 1**.

(B) The same set of RNA templates, as Figure 3B, was used to define the minimum length requirements for the RSV polymerase activities. The reactions were incubated in the presence of NTPs (GTP, CTP, and UTP each at 1.25 mM and ATP at 50 μM with 5 μCi of [α-$^{32}$P]ATP). The right lane shows the molecular weight ladder. The sequences of the RNA oligos are listed in **Table 1**.
